# Barriers to weight management in pregnant mothers with obesity: a qualitative study on mothers with low socioeconomic background

**DOI:** 10.1186/s12884-021-04243-0

**Published:** 2021-11-17

**Authors:** Fahimeh Mehrabi, Najva Ahmaripour, Sara Jalali-Farahani, Parisa Amiri

**Affiliations:** Research Center for Social Determinants of Health, Research Institute for Endocrine Sciences, Shahid Beheshti University of Medical Sciences, P.O.Box: 19395-4763, Tehran, Iran

**Keywords:** Barriers, Obesity, Low socioeconomic status, Pregnancy, Weight management

## Abstract

**Background:**

Maternal obesity is a public health issue that could affect both women’s and children’s health. This qualitative study aimed to identify barriers to weight management of pregnant women with obesity and low socioeconomic backgrounds.

**Methods:**

The current qualitative study has been conducted using a grounded theory approach by analyzing data collected from in-depth interviews with clients of Tehran’s public health care centers for prenatal care. The criteria for selecting participants were excessive weight gain during the first two trimesters of pregnancy, low socioeconomic status, and willingness to share their experiences. A semi-structured guide consisting of open-ended questions was asked in a private room. Open, axial, and selective coding were applied to the data.

**Findings:**

Four main themes emerged from data, each of which has some subcategories: 1) personal factors (unpleasant emotions and feelings, personal tastes/hobbies, workload and responsibilities, and history of diseases), 2) pregnancy status (unintended and high-risk pregnancy), 3) interpersonal relationships and support (lack of a spouse’s support and unhealthy role modeling of relatives), 4) socio-cultural factors/influences (social norms and values, lack of access to health services, and unreliable information channels).

**Conclusions:**

This study provides an overview of the barriers to the weight management of pregnant women from low socioeconomic backgrounds. The results could help develop appropriate health strategies for low socioeconomic women with obesity. Also, health care providers for this group of women could use these findings as a guide to consider their conditions and background.

## Introduction

Obesity in women of childbearing age has become an epidemic in recent decades. The prevalence of obesity in women is reported to be 15% worldwide [1] and 24.1% in Iran [2]. Do not only the repercussions of this complication affect women’s health but also extend across generations. In addition to some severe maternal obesity outcomes, including gestational diabetes, congenital fetal anomaly, miscarriage, dysfunctional labor, pre-eclampsia, and stillbirth [3–5], the long-term influences on offspring are a wake-up call. It is evident that obesity in pregnancy leads to an increased risk of obesity, coronary heart disease, stroke, asthma, type 2 diabetes, and neurodevelopmental disorders in children [6]. In this regard, tackling childhood obesity has been considered one of Iran’s most important priorities throughout recent years; however, existing evidence indicated a knowledge gap in the field of weight management of pregnant women and children under 6 years old in the related national program [7].

Although obesity and lifestyle-related diseases in women are on the rise worldwide, the risk factors and barriers to weight management are not the same in different cultures and socioeconomic statuses [8, 9]. It has been mentioned frequently that Middle-Eastern women are encouraged to have an obesogenic lifestyle [10]. On top of that, financial limitations could make the situation worse. The high cost of prenatal care and lack of adequate knowledge or access to free health services make proper weight management unachievable for low socioeconomic pregnant women [11]. Moreover, the decline in childbearing rates among educated couples indicates the significance of providing adequate care for expectant mothers in low socioeconomic groups, as the majority of next generations are the offspring of this group [12].

Maternal obesity rates in Iran indicate shortcomings in current pregnancy health services [13], especially for low socioeconomic mothers who do not have access to private care. However, research on gestational obesity in Iran has not been focused on weight management problems of mothers from low socioeconomic backgrounds [14, 15], while identifying these mothers’ needs, recognizing their exact situation, and directly hearing their issues could help eliminate defects of maternity care services. Therefore, the present qualitative study aimed to identify barriers to weight management in women from low socioeconomic backgrounds.

## Methods

### Participants and data collection

The current qualitative study has been conducted using a grounded theory approach. The criteria for selecting participants were excessive weight gain during the first two trimesters of pregnancy, low socioeconomic status, and willingness to share their experiences. Participants were among pregnant mothers who received their prenatal care services from public health care centers of Tehran. The main researcher communicated with potential participants to explain the intentions and process of the current research. If the participants agreed to partake in the study, an interview was scheduled. To acquire participants’ views, 25 in-depth interviews were conducted from August 2018 to February 2020. An interview protocol was developed, including a list of interview questions, the procedural level of interviewing, and a script of what should be said before and at the end of the interview, prompts for the interviewer to collect informed consent, and the essential demographic information. All of the interviews, conducted by the main researcher, lasted between 1 and 3 h. A semi-structured guide consisting of open-ended questions was asked in a private room, enabling respondents to sufficiently explain their personal opinions, perceptions, and experiences. First, each participant was asked to describe their perceptions of and experiences with the main factors determining their excessive weight gain process and the main barriers to proper weight management during pregnancy. All interviews were conducted, audiotaped, transcribed, and analyzed in Persian. A qualified bilingual individual, adept in the nutritional qualitative research’s professional terminology, validated the translation quality and conceptual equivalence to establish credibility.

The ethics committee of the Research Institute for Endocrine Sciences, Shahid Beheshti University of Medical Sciences, approved the study. Participants provided written informed consent before the interviews, and explicit permission was sought for audiotaping.

### Data analysis

Based on the grounded theory approach, data collection and analysis were done simultaneously and through constant comparative analysis in the current study [16]. Open, axial, and selective coding were applied to the data. During open coding, each transcript was reviewed by at least two authors, and the data were modified to codes. Variations in coding were resolved during discussions. Conceptually similar codes or those that were related in essence were classified into subcategories. Axial coding was done in order to clarify how the emergent subcategories were associated with preliminary categories. Analytical tools that included asking questions and making comparisons were used to find the characteristics of each concept. Interviewing was stopped when data saturation occurred: when no more codes were identified during the last couple of interviews and when the emerged categories were “sound.”

In this study, the data’s credibility and confirmability were established by prolonged, in-depth engagement with participants and member checking. Choosing participants from low socioeconomic backgrounds with different age groups, educational levels, and occupations contributed to a richer variation of the phenomena under study. To confirm dependability, three other researchers scrutinize the study. Results were also checked with some of the expectant mothers, who participated in the research, and they confirmed the fitness of the results as well. All research details, including procedures, actions, and decisions, were documented for auditing purposes.

## Findings

**Fig. 1:**
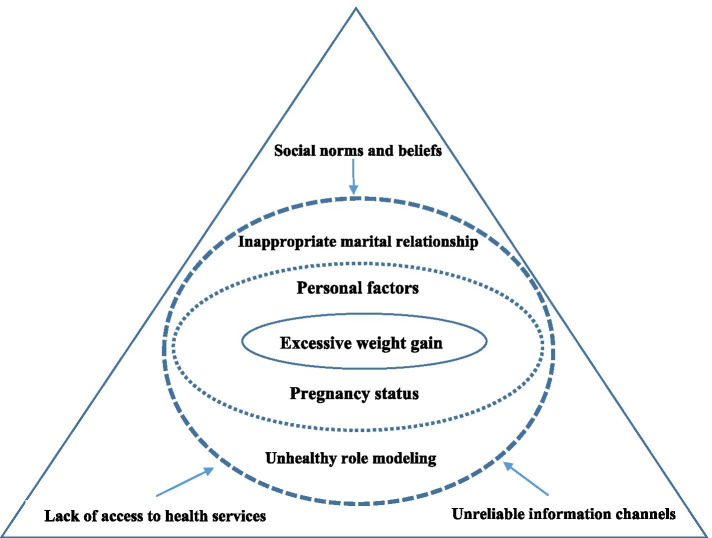
Conceptual framework of barriers to weight management in pregnant mothers

A heterogeneous group of pregnant mothers with different employment, education, and reproductive histories participated in the study (Table 1). Figure 1 presents the final conceptual model that explains the main barriers to a healthy lifestyle that could lead to improper weight management in pregnant mothers. The main themes that emerged from data include (i) Personal factors, (ii) Pregnancy status, (iii) Interpersonal relationships and support, (iv) Socio-cultural factors/influences.

**Table 1:** Characteristics of study participants

Variable	Number	Percent
Age (year)		
20–30	5	20
31–41	19	76
Number of pregnancies		
Nulliparous	7	28
Multiparous	18	72
Abortion history		
Yes	5	20
No	20	80
Education		
Primary	8	32
Secondary	8	32
Higher	9	36
Job-status		
Housewife	22	88
Employed	3	12
Spouse education		
Primary	6	24
Secondary	8	32
Higher	11	44
Spouse job status		
Employed	1	4
Unemployed	24	96

Personal characteristics included four subcategories: unpleasant emotions and feelings, personal tastes/hobbies, workload and responsibilities, and history of diseases. Pregnancy characteristics encompassed two subcategories: unintended and high-risk pregnancy. Interpersonal relationships/support included two subcategories: lack of a spouse’s support and unhealthy role modeling of relatives. Socio-cultural factors/influences included three subcategories: social norms and values, lack of access to health services, and unreliable information channels.

### Theme 1. Personal factors

#### Unpleasant emotions and feelings

In the current study, a group of mothers noted that their unpleasant feelings such as sadness, anger, fear, disgust, anxiety, and loneliness affected their lifestyle and consequently weight management during pregnancy. In this regard, a mother pointed out the loss of her daughter and the burden of grief as the main influential factor on her eating habits: *“I always feel a big sadness in my heart, three years ago my eldest daughter got cancer and died at the age of 14. I was mourning and had no motivation for living. Sometimes I overeat and sometimes have no appetite to eat” (33 years old, multiparous mother).* Accordingly, another pregnant mother described her anxiety and its repercussions on her dietary intake: *“Sometimes when I am nervous, I could not eat anything for three or four days, but then I eat a lot” (32 years old, multiparous mother).* Based on the mothers’ statements, fear was another emotion that could affect their eating habits during pregnancy, especially in those who were a new bride or expecting their first baby: *“I’m afraid of low blood pressure. My sister felt weakness during her pregnancy and once lost her consciousness. I don’t want to have a similar experience; so I eat a lot” (29 years old, nulliparous mother).* Accordingly, some mothers pointed out loneliness as a determinant of their lifestyle: *“Most of the time I am alone, it has been five months since we immigrated and I have not seen my relatives. I try to entertain myself by cooking and eating” (29 years old, multiparous mother).* Finally, some participants referred to the hate as another influential experience: *“My husband and I have to live with my husband’s mother. Once, she told me that I am not allowed to cook in the house. Although my doctor advised me to eat more protein, I always have to leave the table hungry and instead eat junk foods such as biscuits in my room because I don’t want to see her. I hate her, and I would like to die and not see her; unfortunately, due to poor nutrition, I developed gestational diabetes and became very obese.” (29 years old, nulliparous mother).*

#### Personal taste/ habits

Some pregnant mothers pointed out overeating as the leading cause of their excessive weight gain during pregnancy: “*I think I eat a lot. I can’t stop eating anything I find at home. (33 years old, multiparous mother).* This overeating has been observed for some particular foods: *“I eat a lot of bread. I am very interested in Lavash bread (a kind of traditional Iranian bread) that I even ate that with rice and pasta, I know it is not good but I can’t help it” (30 years old, multiparous mother*) and could be intensified during specific activities: *“I eat more and more sweets every day, especially while reading a novel or watching T.V.“ (24 years old, multiparous mother).* Another participant emphasized her taste as the important factor for choosing high-dense calorie foods and increasing the risk of overweigh: *“I like greasy foods. In general, my family and I hate dry food. I believe that oil-free food is for sick people, and I don’t want to eat it” (39 years old, multiparous mother).*

#### Workload and responsibilities

In the current study, some pregnant mothers attributed their unhealthy eating habits to their job: *“I work 18 hours a day standing up. I do not have time to eat and sleep at all because of the customers. I work with chemicals hair colors that smell bad, especially since I got pregnant. So, when I get home, I feel starving and eat a lot and, I fall asleep right away” (32 years old, multiparous mother).* Another mother complained about her heavy and challenging job, which could affect her lifestyle and result in excessive weight gain: *“I am in charge of cleaning a sports club. I have to clear the whole area of the club and wash the bathrooms and toilets and carry the heavy garbage every day. So, I become famished in the evening. When I go home every day, I must eat 5-6 eggs fried in butter or jam and butter with two bread loaves. At the end of the night, when my husband comes, I also have dinner with him “(34 years old, multiparous mother).*

### Theme 2. Pregnancy status

#### Medical condition and background

Aside from the primary factors that emphasized mothers’ unhealthy lifestyle as the main determinant of their overweight during pregnancy, some of the mothers pointed out the pivotal role of their sustained genetic backgrounds: *“I have been very obese since childhood, like my mother’s family who are all obese” (24 years old, nulliparous mother).* In addition, those participants who had experienced a delay in pregnancy attributed their overweight to potential hormonal imbalance, which could negatively influence their weight management efforts: *“We wanted a baby, but my prolactin hormone was high, I couldn’t get pregnant. My doctor said that your weight gain was due to hormonal changes. I told myself that whatever I did to lose weight was useless” (33 years old, nulliparous mother).* This situation was aggravated by infertility treatments: *“My weight gain is due to the hormonal injections prescribed to treat my infertility. Doctors said weight gain would be normal in this condition” (24 years old, nulliparous mother).* Further to hormonal effects, passing a high-risk pregnancy also exacerbated factors such as a sedentary lifestyle and increased rest time: *“I was completely resting during my pregnancy because I was undergoing IVF treatment and under the supervision of a medical team. Recently a nutritionist has joined because I have gained a lot of weight. Of course, I am in the last week, and I have swelling. Maybe that’s why.” That I don’t lose weight.“ (24 years old, Nulliparous mother). “The condition of the fetus was unsuitable. I saw spots twice. I have completely rested from the first months and gained a lot of weight” (30 years old, Nulliparous mother).* Besides high-risk pregnancy, some mothers referred to their medical conditions as the main determinants of excessive weight gain during pregnancy: *“I was thin before marriage. After marriage, I had severe headaches due to a series of mental and nervous problems, and then MRI revealed that my spinal channel was narrow in my neck. And I used melatonin because of my heart palpitations, so I was sleepy, and I had to overeat before taking my pills. Although I could prevent my stomachache, I gained 35 k” (33 years old, nulliparous mother).*

#### Unintended pregnancy

In the current study, a considerable number of mothers stated that they became pregnant unintentionally and lost their motivation to lose weight. This unpleasant situation distorted their diet, sleeping patterns, and daily activities, which resulted in more weight gain: *“Before I got pregnant, I weighed about 97 kilos, but now my weight is 115 kilos. I am not motivated to lose weight. To be honest, I intentionally did not eat properly to make the baby abort” (32 years old, multiparous mother).* One of the mothers said: *“Honestly, I did not intend to get pregnant quickly because my son is only 11 months old. I wish I got pregnant again at least five years after my first pregnancy because I was very overweight, and now I am pregnant again” (33 years old, multiparous mother). “I have two older daughters, and I did not expect to be pregnant again, but unfortunately, I got pregnant, and it was unintended pregnancy. At first, I tried very hard to have an abortion, but my doctor talked to me a lot. My conscience bothered me, and my husband did not want to; I am no longer able to lose weight after giving birth” (37 years old, multiparous mother).*

### Theme 3: inter-personal relationships/support

#### Inappropriate marital relationship

In the current study, a number of participants referred to their marital dissatisfaction as the most critical barrier to weight control during their pregnancy. In this regard, some of them complained about their husbands’ ignorance and misbehavior, which have mainly been rooted in their different cultural backgrounds and resulted in motivation loss to a healthy lifestyle and weight control: *“I have a lot of cultural differences with my husband. I was interested in studying and exercising, but my husband only cares about eating, and after a while, I became an obese woman who only cooks well. I have no motivation anymore” (37 years old, multiparous mother).* Another mother spoke of her husband’s constant disregard for her: *“My husband doesn’t see me at all, even if he sleeps next to me. It doesn’t matter if I’m fat or thin, happy or sad, I’m indifferent to him, and I’m having fun with the kids and cooking, and my pleasure is cooking delicious food and eating”. (30 years old, multiparous mother).* Another mother said about strict rules set by her husband for doing outdoor activities as a result of his family’s traditional attitudes: *“I would like to go to a good gym with a good coach to supervise my activities; however, my husband does not allow me and says, “Go to the park.”. Surprisingly, when I want to go to the park for walking, he says, “I said something, why did you take it seriously? Does a noblewoman go to the park alone? This manner comes from his inappropriate beliefs” (33 years old, nulliparous mother).* Another mother said her husband did not support her financially: *“My husband is very stingy. If I need anything, I have to tell him many times to prepare it, especially the food ingredients, so when I go to my mother’s house, I crave a lot of food” (24 years old, multiparous mother).* Another mother said her husband insisted that she eat nutritious food during pregnancy: *“My husband is thrilled to be a father soon. He keeps telling me to eat walnut kernels, almonds, and figs and nutritious foods, and I’m getting heavier by the day*” (33 years old, multiparous mother). *“My husband also forces me to eat nutritious foods, and I get fatter day by day” (33 years old, multiparous mother).* Some mothers attributed their weight gain and obesity to their husbands desires: “*My husband likes my face to be fat, but whatever I eat, my body gets fat, but my face is thin. Now that I am pregnant, my face is swollen and he says you are magnificent” (33years old, nulliparous mother).* Similarly, another mother stated: *“My husband hates a thin woman, everyone says I want you to be fat and plump, then when he wants me to be like this, why torment myself, I can hardly lose weight” (42 years old, multiparous mother).*

#### Unhealthy role modeling by mothers and friends

Most participants mentioned family and friends’ role as one of the most important underlying factors for their unhealthy eating habits. Some mothers believe that they eat more when they are with relatives and friends. One group attributed the cause to a sense of cheerfulness in communicating with loved ones. Another group said that others insisted on encouraging them to binge eating and reducing the activity: *“Honestly, when I’m with my family, I always eat and sleep, and I am pleased” (33 years old, multiparous mother).* Another mother spoke of her friend’s advice: *“I have a friend who tells me: “You are pregnant, and you have to eat as much as you want” (33 years old, multiparous mother)*. It can be said that mothers have the most significant impact on their pregnant daughters’ nutrition patterns compared to other people. *“Cooking for me is desired by my mother. Since I have been pregnant, she has constantly been cooking delicious food for me. (33 years old, multiparous mother). “My mother says that natural food is the best. I used to buy dairy from the store, but now she makes cheese, yogurt, sheep’s butter, and sends it. I also use them, especially butter” (24 years old, multiparous mother). “My mother says that local oil is suitable for a pregnant woman, and you should eat it every day “(24 years old, multiparous mother).*

### Theme 4. Socio-cultural factors/influence

#### Social norms and beliefs

Besides personal and interpersonal characteristics that could affect weight management during pregnancy, some socio-cultural factors influenced mothers’ weight status. In this regard, some of the mothers referred positive attitude toward obesity which lead to a lack of desire to control weight: *“In our city, this body shape is a sign of beauty and physical strength, and people do not label it as obesity “(33 years old, multiparous mother)* and another participant stated*: “Everyone says when the baby is a girl the body will be swollen, and you will look obese” (24 years old, multiparous mother).* Also, some beliefs influenced participants’ food intake and eating habits, resulting in overeating during pregnancy. In this regard, a mother said: *“Every time I have a stomach ache, I drink soda to digest food, and the pain quickly subsides” (32 years old, multiparous mother)* and accordingly another pregnant mother said: *“I have to eat a lot of fruit so that my body absorbs more vitamin because too much vitamin is perfect for the growth of the baby “(34 years old, multiparous mother).*

#### Unreliable information channels

In the current study, most of the participants were dependent on the internet and browsed various websites to find answers for their questions: *“To search for anything during pregnancy, I search the internet to make sure it’s not bad for my baby” (34 years old, multiparous mother).* Also, another participant stated that: *“During my pregnancy, many people told me to eat this and not to eat that, but I searched on the internet and ate it if it was useful” (34 years old, multiparous mother).* In some cases, this obtained information was even preferred to specialists’ advice: *“I search for Nini Site a lot, especially about the growth and evolvement baby or nutrition of during pregnancy, and there no need to see a nutritionist anymore.” (37 years old, multiparous mother).*

#### Lack of access to health services

The economic burden and high cost of mental health services and medical services were among the factors that caused the most adverse conditions for most participants, forcing many mothers not to use these services, especially in cases such as expensive clinical services as well as the lack of free psychological counseling services for mothers in this period: *“The cost of a visit to a gynecologist is very high, and we cannot afford it, so I did not go to the clinic from the third month onwards. I went to a nutritionist in my first pregnancy, but now I get fatter because I could not go to a nutritionist. At the same time, some pregnant women in a good financial position are under the care of gynecologists, nutritionists, and dermatologists. They provide good and expensive vitamins and supplements, and this easily and without breaking their body control their weight during pregnancy. They go through times, but I have to endure these conditions, and I cannot lose weight.” (32 years old, multiparous mother).* Another mother said about the importance of having free psychological services during pregnancy: “*I cannot go to a psychologist or psychiatrist because it costs so much. Especially for a mother who is experiencing pregnancy for the first time, it is difficult to accept mental and physical conditions, especially to lose fitness or change her emotions, and improving my mental condition helps to improve my physical condition; even our husbands need psychological services on how to treat their wives during pregnancy properly” (37 years old, multiparous mother).* Another participant spoke of non-compliance with health insurance obligations: *“Unfortunately, health insurance does not cover most of the necessary medications and genetic testing, and it puts a lot of pressure on the family” (42 years old, multiparous mother).*

## Discussion

The current study aimed to explore barriers to control weight gain during pregnancy in mothers with low socioeconomic backgrounds. Our results showed that the most prominent barriers faced by pregnant mothers could be considered into three individual, interpersonal, and community levels. Based on the participants’ statements, at individual and interpersonal levels, mothers’ unpleasant emotions, as well as inappropriate marital relationships, were the most critical factors contributing to excessive weight gain during pregnancy, which were affected by social norms and beliefs, access to health services and information.

Regarding individual factors that could affect the weight gain process during pregnancy, the current results revealed factors related to both mothers’ characteristics and pregnancy status. Our findings showed that unpleasant emotions, eating based on appetite, and high workload as the predominant barriers to proper weight management in pregnant mothers at the individual level. These results are consistent with previous studies that suggested the determinants mentioned above as influential factors on expectant mothers’ lifestyles [17]. Based on the current results, women faced periodic overeating and anorexia due to their aroused overwhelming feelings or gorging as a response to loneliness and the fear of weakness during pregnancy. Although eating in response to unpleasant emotions, not actual hunger, is an ineffective and common coping strategy [18], women in the lower socioeconomic class are at higher risk of using unhealthy mechanisms for emotion regulation due to lack of access to education and psychological services and higher stress levels than women with upper-income [19]. In addition to emotional eating, our findings indicated eating based on appetite in some pregnant participants, which often led to gorging high-density calories and unhealthy foods. Many prior studies have mentioned the lack of self-control and cravings as a primary cause of excessive gestational weight gain, in line with our results [11, 20]. However, little evidence supports the role of hormonal fluctuations, nutritional deficits, or needs of the developing fetus in developing food cravings in expectant mothers [21]. In this regard, it should be noted that some common beliefs in society reduce pregnant women’s motivation for managing weight and self-control by normalizing excessive weight gain during pregnancy [22]. Also, findings of a recent study among Dutch population showed cravings for high-fat foods works as a mediator in the association between appetite in response to emotional cues and excess gestational weight gain [23]. Finally, long working hours a day in manual labor were cited as another obstacle to proper weight management by the current study participants. Other investigations have also considered a high workload as a barrier to managing nutritional intake and prenatal care previously [24]. Despite the low socioeconomic background of the current participants, just a minority of them had to be employed due to financial difficulties. Considering traditional Middle-Eastern culture in which breadwinning is generally men’s responsibility [25], employed pregnant women experienced higher psychological pressure, leading to their unhealthy lifestyle and excessive weight gain. In contrast, in a qualitative study, low-income American women cited unemployment as a psychological nuisance and a barrier to lifestyle modification.

In addition to personal characteristics, some of the current participants attributed their excessive weight gain to their pregnancy status. Pre-pregnancy overweight and the related hormonal imbalance were mentioned by current expectant mothers as obstacles to weight management. Consistent with our findings, a large body of evidence has been written on the adverse effects of pre-pregnancy overweight and its relation to women’s hormone profile and reproductive performance [26]. Also, bed rest due to high-risk pregnancy status and dramatic decrease in physical activity levels could increase emotional difficulties leading to more need for mental health services [27]. Moreover, women who experienced an unintended pregnancy were the most disappointed group to manage their weight. In agreement with our findings, a comparative study in Iran showed that compared to women with planned pregnancies, women with unwanted pregnancies had lower scores for physical and mental health status and self-care behaviors such as using supplements, vaccination, and nutrition [28]. A global investigation found that the rate of unwanted pregnancies in developing countries from 1999 to 2014 was substantially higher than in developed countries [29]. Considering that Iran is among countries with restrictive laws about abortion [30] and people with low income are more likely to experience an unplanned pregnancy [31], an essential obstacle to establishing a routine with healthy habits in the lower socioeconomic status could be unintentional pregnancy.

Beyond individual factors, our results revealed the crucial effects of husbands, families, and friends on pregnant women’s lifestyles and weight management. Previous research has also shown that as spouses and people around expectant mothers could play a motivating role in leading a healthy lifestyle, they could negatively affect their nutritional habits and physical activity [32, 33]. According to the current findings, expectant fathers’ preferences regarding their spouses’ body shapes were a direct deterrent to weight management during pregnancy. Most pregnant mothers in the present study frequently mentioned their husbands’ interest in chubby women, which resulted in constant encouragement of overeating and excessive weight gain. Consistent with our findings, previous studies mentioned that men with fewer financial resources prefer heavier women [34]. Regarding indirect effects, marital relationships based on patriarchal beliefs affect women’s lifestyles and weight management by depriving them of decision-making and marital discontent through deteriorating women’s feelings. In this regard, women financially dependent on their husbands have to remove valuable and healthy plans without their support. Besides, even free activities are subject to husbands’ permission. Moreover, marital dissatisfaction has been recognized as an essential determinant of psychological conditions during pregnancy, influencing diet and activity. As a longitudinal study among American couples showed significantly greater fluctuations in low-income couples’ marital satisfaction [35], and a recent study in Iran indicated the adverse effect of marital dissatisfaction on the nutrition of expectant women [36], lower socioeconomic women could be more derived to unhealthy eating habits. In addition to spouse influence, study participants stated that their families and friends continuously encourage them to indulge in eating and resting. Their advice, based on the belief that overeating and sleeping make both mother and infant healthier, has influenced pregnant women’s lifestyle and has driven them to overeat and reduce physical activity. Similar to our results, other studies have shown that misconceptions about pregnancy health develop a significant barrier to proper weight management and reinforce harmful eating habits in pregnant women [37].

Regarding a broader extent than personal characteristics and interpersonal relationships, socio-cultural factors including social norms and beliefs, unreliable information channels, and lack of access to health services were identified as impediments to the weight control of pregnant women in the present study. As participants stated, their cultural context and some misconceptions about prenatal care led them to an obesogenic lifestyle. Our findings are consistent with the socialized actor theory, which assumes norms influence individuals’ choices by shaping their needs and preferences [38]. While traditional cultural restrictions for women in the Middle Eastern countries drive them to excessive weight gain [10], and obesity is a sign of health for some Iranians [39], not surprisingly, most expectant mothers in the present study see no need to weight control. A comparative study has well illustrated the effects of cultural differences on exercise during pregnancy. It revealed that Australian pregnant women are more physically active than their Chinese counterparts since some china beliefs recommend expectant mothers not to walk throughout pregnancy [40]. Aside from some social norms, inaccurate information sources could affect mothers’ lifestyles. In this regard, previous studies also emphasized the high amount and importance of internet usage for seeking health-related information during pregnancy [41, 42]. A study showed that although the internet is the second consultant for reaching requisite knowledge of prenatal care among low socioeconomic women, this population cannot use it for their benefit properly and find reliable sources [43]. In addition, a review study noted that most pregnant women who follow internet websites for seeking information do not ask health professionals about the accuracy of the content [44]. Our findings emphasized the lack of access to appropriate medical and psychological services for mothers from a low socioeconomic background. In agreement with our results, providing accessible or affordable health services for low-income pregnant women has been suggested before [17, 45].

This is one of the first qualitative studies aimed to explore the main barriers to weight management in pregnant mothers with low socioeconomic backgrounds. The current findings make an excellent opportunity to understand expectant mothers’ perceptions and experiences regarding the mentioned barriers in different personal, inter-personal and socio-environmental levels in an Eastern-Mediterranean context. Volunteer participation, as well as pregnancy circumstances, resulted in some data gathering limitations. Also, selecting mothers from urban areas of Tehran does not reflect pregnant mothers’ experiences residing in suburban and rural areas. Further research is recommended in these areas.

## Conclusion

This study provides an overview of the barriers to the weight management of pregnant women from low socioeconomic backgrounds. Based on the conceptual framework that emerged from the current data, the most critical barriers to weight management in pregnant women from the lower socioeconomic classes are rooted in various factors at the individual, interpersonal and environmental-social levels. In this regard, careful attention to the most modifiable factors at all mentioned levels in the form of ecological models seems necessary. The present results can be the basis for designing the tailored models and, consequently, comprehensive programs to promote women’s health and safe pregnancy among vulnerable groups in Iran and similar societies.

## Data Availability

The datasets used and/or analyzed during the current study available from the corresponding author on reasonable request.
